# Barriers and Facilitators to Accessing Digital Health Tools Faced by South Asian Canadians in Surrey, British Columbia: Community-Based Participatory Action Exploration Using Photovoice

**DOI:** 10.2196/25863

**Published:** 2022-01-13

**Authors:** Antonia Hyman, Elizabeth Stacy, Humaira Mohsin, Kaitlin Atkinson, Kurtis Stewart, Helen Novak Lauscher, Kendall Ho

**Affiliations:** 1 Digital Emergency Medicine Department of Emergency Medicine, Faculty of Medicine University of British Columbia Vancouver, BC Canada

**Keywords:** immigrants, community-based participatory action research, eHealth, delivery of health care, photovoice, South Asian, digital health, mobile phone

## Abstract

**Background:**

South Asian community members in Canada experience a higher burden of chronic disease than the general population. Digital health innovations provide a significant opportunity to address various health care challenges such as supporting patients in their disease self-management. However, South Asian community members are less likely to use digital tools for their health and face significant barriers in accessing them because of language or cultural factors.

**Objective:**

The aim of this study is to understand the barriers to and facilitators of digital health tool uptake experienced by South Asian community members residing in Canada.

**Methods:**

This study used a qualitative community-based participatory action research approach. Residents from Surrey, British Columbia, Canada, who spoke 1 of 4 South Asian languages (Hindi, Punjabi, Urdu, or Tamil) were invited to participate in focus group discussions. A subsample of the participants were invited to use photovoice methods in greater depth to explore the research topics.

**Results:**

A total of 197 participants consented to the focus group discussions, with 12 (6.1%) participating in the photovoice phase. The findings revealed several key obstacles (older age, lack of education, and poor digital health literacy) and facilitators (social support from family or community members and positive attitudes toward technology) to using digital health tools.

**Conclusions:**

The results support the value of using a community-based participatory action research approach and photovoice methods to engage the South Asian community in Canada to better understand digital health competencies and needs. There were several important implications for policy makers and future research, such as continued engagement of community leaders by health care providers and administrators to learn about attitudes and preferences.

## Introduction

### Background

The South Asian population is one of the largest and fastest-growing ethnic minority groups in Canada, comprising nearly 2 million individuals (5.6% of the general population) in 2016 and increasing annually [1]. South Asian immigrants (ie, people whose ethnic roots originate from the Indian subcontinent, including India, Pakistan, Sri Lanka, Nepal, Bangladesh, Maldives, and Bhutan) experience a higher burden of chronic diseases than the general population (eg, cardiovascular disease and type 2 diabetes) [2,3], tend to have more risk factors for chronic diseases [4,5], and face many challenges accessing tools to prevent chronic disease and manage their health conditions [6,7]. Consequently, there is a pressing need to address barriers to health care access for South Asian community members [8] and reduce health inequities [9].

### Digital Health Tools

The prevalence and widespread availability of digital health (eHealth) innovations provide a significant opportunity for addressing various health care challenges [10]. A systematic review of literature on *eHealth* attempted to understand the various ways it can be defined and found 51 different definitions [11]. The authors of the review concluded that developing another definition in an attempt to improve or summarize previous definitions did not make sense, given that specific wording will have a place in different contexts, and therefore did not merit delineating further. For the purposes of this research, digital health can be broadly defined as the transfer and delivery of health care and health resources and services through information and communication technologies [12]. Digital health *tools* specifically are considered the physical tools required to access digital health technology. For example, the use of electronic health records, making medical appointments or filling prescriptions on the web, telehealth for rural patients, or using a smartphone app to track one’s health status. It is estimated that if all Canadians had access to digital health tools, it would decrease 47 million in-person visits to health care providers and 18.8 million hours in absenteeism from work, representing an annual gain of CAD $400 million (US $312 million) in gross domestic product, and provide 51 million additional hours to spend on leisure activities [13]. Research on digital health and the impact on quality of care tends to measure quality of care from the health care professional or management perspective. For instance, a large retrospective study compared quality of care at sites using electronic health records versus those using only paper-based records for patients with chronic health conditions [14]. The study found an improvement in quality of care and outcomes according to regional standards of care. The successful incorporation of digital health tools into one’s health care activities relies on appropriate levels of digital health literacy [15], which is based on the intersection of six foundational literacies: traditional literacy and numeracy as well as computer, health, information, media, and science literacies.

Despite the benefits, there can be significant barriers to the uptake of digital health tools by individuals, such as sociotechnical challenges [16] or misalignment between design and user needs [17]. In particular, immigrants and older adults can face specific challenges concerning access and uptake of digital health tools [12]. In a sample of Punjabi-speaking South Asian individuals in Canada, older age, female gender, lack of language proficiency, and lower socioeconomic status were all associated with less use of technology for health self-management [12]. A large survey conducted in Alberta, Canada, gives a picture of technology and digital health tool use among South Asian Canadian adults. It found that 74.5% of the participants reported using the internet, with 47.8% using it for health information–seeking purposes; 74.9% reported using smartphones, and of these respondents, 30.7% had apps related to health and fitness [18]. The survey also found that older age and lower educational attainment predicted lower use of the internet and smartphones, among other factors. In addition, the survey found that preferring languages other than English predicted lower likelihood of using different forms of eHealth. Although digital health innovations exist to support prevention and management of chronic diseases, these tools are often not culturally tailored or used by South Asian populations in Canada [12,19]. This gap could lead to disparities in health knowledge and services [20]. As such, implementing digital health–related innovations among South Asian Canadians requires understanding and addressing barriers and facilitators that they may face when using digital health tools.

### Community-Based Participatory Action Research and Photovoice

Community-based participatory action research (CBPAR) methods [21,22] have several advantages compared with conventional researcher-driven methods. By co-designing and co-leading the advancement of research between community members and researchers, CBPAR can empower and build capacity in the community and acknowledges that community members are experts in their own right concerning issues affecting them [23]. Thus, CBPAR can foster understanding of research from the participants’ point of view and capture important insights that may be missed by investigators external to the community.

Photovoice is a process that uses photography and discussion to bring attention to community issues and aims to empower the community of interest [24,25]. This methodology invites participants to take photographs in their daily lives that capture concepts important to the research question [26], and they write the meaning of the photograph in their own words. Once the set of photographs is captured, a group discussion follows to share the meaning behind the photographs. A review of studies using photovoice illustrates that this approach can engage communities in meaningful research to promote positive change, even in hard-to-reach populations [23]. Photovoice can overcome potential barriers related to language and literacy through the emphasis on photographs [26], and therefore it was identified as a valuable method for this study where such barriers may exist among participants.

### This Study: Interactive Health Education Action for Life (iHEAL)

We conducted this 2-phase CBPAR study to better understand the barriers to and facilitators of digital health uptake among the South Asian Canadian community. This study engaged the South Asian community in Surrey, a municipality in Metro Vancouver in western Canada with a large South Asian population. At the time of data collection, Surrey had a total population of 517,885, with a growth rate of 11% from 2011 to 2016 (compared with 7% for Metro Vancouver) [27]. Immigrants make up 43% of the population, with 41% originating from India, and South Asian individuals make up one-third of the population [27]. The regional health authority has identified that South Asians have poorer reported health than the general population, with type 2 diabetes and cardiovascular disease 2-3 times higher and 46% of the older adults having ≥2 chronic diseases [5].

### Research Aims

This iHEAL study has 3 primary objectives. First, it aims to gain a deeper understanding of the barriers and facilitators experienced by Surrey’s South Asian community in using digital health tools for self-management and prevention of chronic disease. Second, it aims to provide an opportunity to engage the community and increase awareness and capacity with research and technology. Third, it aims to illuminate key policy areas related to the development and uptake of digital health tools in this population.

### Ethical Approval

All procedures performed with participants in this study were in accordance with the ethical standards of the Behavioural Research Ethics Board of The University of British Columbia, which provided ethics approval for conducting the study (H14-02308), and with the 1964 Declaration of Helsinki and its later amendments. Informed consent was obtained from all individual participants included in this study.

## Methods

### Study Design

This iHEAL study comprised two phases: (1) focus group discussions and (2) photovoice. Given the diversity within Surrey’s South Asian community and the different languages spoken, the methods purposively targeted communities speaking Punjabi, Hindi, Urdu, and Tamil to represent a plurality of views [27]. Guided by community-based participatory action methodology [21,22], the team established an advisory committee to expand the engagement of community organizational leaders as well as to recruit 7 peer community researchers (PCRs) from the South Asian community to provide culturally appropriate research support for the project and build research capacity in the community itself.

The research team sought to ensure that the initiative was truly co-designed and co-led with the community. The research group had a long-standing relationship in Surrey’s South Asian community, with multiple researchers on the team being from the community itself. These researchers came with a wide variety of perspectives and expertise. The initial phases of the initiative were informed by two groups: (1) a broad group of stakeholders with various interests, agendas, and mandates and (2) leaders from various community-based organizations. Both groups aimed to collaboratively identify key research questions, target populations, methods of engagement, and other aspects core to the research initiative. These sessions were facilitated in the language of the community by members of the community. The research tools developed with partners were translated into the target languages, after which a small cadre of partners reviewed them to ensure that the questions made sense and resonated. For instance, a question regarding any differential impact of gender roles and experiences of digital health tools was included because this was identified as an important area to explore by the community. The community partners conducted focus groups, facilitated photovoice sessions, analyzed data, and disseminated findings.

### Participants and Sampling Method

Recruitment was facilitated by a community liaison, community leaders, PCRs, and The University of British Columbia research team. Language-appropriate posters and recruitment booths were hosted by the team at community centers, schools, and places of worship. Eligible participants were adults (aged ≥18 years) of self-identified South Asian background who were able to converse in 1 of the 4 target languages (Punjabi, Hindi, Urdu, or Tamil) or English. Taking into account the population who face the highest barriers and challenges to integrating digital health tools into their self-management regime [12,18], recruitment primarily targeted older adults. However, the inclusion criterion was kept at age ≥18 years to accommodate other participants interested in joining the study or younger family members of participants interested in joining the study. Notably, older adults are often supported in their technology needs by other family members in South Asian households. Interested individuals were provided with further information about the study and given time to consider and voice any questions or concerns, after which they could give informed consent if they wished to participate.

### Procedures and Data Collection

#### Phase 1

Focus group discussions were semistructured, with questions designed to explore barriers to and facilitators of digital health tool use. A trained focus group moderator led each discussion using a template with 5 primary questions and follow-up questions where clarification was necessary (Textbox 1). Each focus group discussion was 60 to 90 minutes in duration and audio recorded with participants’ consent. The 7 PCRs guided the focus group discussions and provided language support as needed.

**Primary questions for the phase 1 focus group discussions.**

**Primary questions and possible follow-up questions and prompts**

- What is one important health related priority you have for you and your family?
  What are some of the obstacles you come across in staying healthy? Think of any and all aspects of health such as physical health and mental health.
- How do you most commonly access the health information you need?
  Why do you choose to use those resources in particular?
  Whom do you trust most when deciding what information will help you manage your health?
- Does technology play a role helping you manage your health?
  What do you use technology to help with specifically?
  What stops you from using technology?
  What makes some technologies easier or harder to use than others?
- What has your experience been with health information on the web?
  Do men and women experience the same challenges to using technology to obtain health information?
  What do people see as concerns and barriers (for example, is the material available in your own language?)
- How is the current health system supporting or interfering with your ability to manage your health?
  How can the health system support you to use digital health technology to better manage your health?

#### Phase 2

We sought to recruit 12 participants from phase 1 into the subsequent photovoice phase, given that this sample size is the *gold standard* for the photovoice process and ensures a proportional selection of participants from each language community [21,22]. Orientation sessions were held to review the study purpose, time commitment, and procedures, as well as to answer questions. The sessions explained how to use the cameras provided and gave participants the opportunity to practice taking, accessing, and deleting photographs. Each participant was assigned a PCR to provide support for the duration of this phase. Participants were given 14 days to take 10-15 photographs that reflected their experiences of using technology to improve their own health and the health of their community. Three discussion questions to guide the photography activity were developed by the researchers and PCRs to ensure cultural appropriateness:

1. What comes to mind when you think about using technology to learn more about health or manage it?
2. What difficulties do you face when using technology to learn more about health or manage it?
3. What helps you use technology to learn more about health or manage it?

The photographs were printed and used in the focus group discussions with the consent of participants.

#### Data Analysis

The focus group discussions from both studies were professionally translated and transcribed verbatim. A constant-comparison method [28-30] was used to analyze the focus group transcripts using NVivo software (version 10; QSR International). Anonymized data were uploaded onto NVivo, and the data from all focus groups were compiled and, for each phase, analyzed separately in 3 stages. Stage 1 involved chunking the data into smaller units and coding them into a few words or short paraphrased text according to the essence of each individual quote. Stage 2 involved grouping these codes into wider categories, and stage 3 involved regrouping or merging related text to create overarching themes. This approach is useful when analyzing data across multiple focus groups, enabling the comparison of data across groups to see if similar themes emerge and check for saturation of data to indicate the salience of themes across the entire sample. The data were independently analyzed by 2 researchers (KA and HM), and themes were checked and cross-referenced by a third researcher (AH) to determine reliability and validity. The final themes were selected based on consensus and the quantity and quality of supporting data. A researcher involved in the data analysis was from the South Asian community and provided additional insight to contextualize any themes that were culturally specific. The themes were presented back to participants at a presentation as part of an event to showcase the photographs taken in phase 2 (with consent), and feedback was obtained from participants indicating that the themes captured the essence of the discussions and did not miss key information.

## Results

### Overview

Phase 1 data were collected from March 2015 to June 2016. A total of 197 individuals residing in Surrey participated: 81 (41.1%) Punjabi-, 67 (34%) Hindi-, 35 (17.8%) Urdu-, and 14 (7.1%) Tamil-speaking participants. In all, 26 focus groups were conducted (mean 7.6, SD 1.8 participants per group), with 13, 7, 4, and 2 focus groups conducted for the Punjabi-, Hindi-, Urdu-, and Tamil-speaking communities, respectively. During the initial focus groups, the moderators observed that men spoke much more than women; therefore, subsequent focus groups were separated by self-reported gender to promote a balance of perspectives. For phase 2, of the 197 participants, 12 (6.1%) completed the photovoice component, and 2 focus groups were conducted in July 2016 to share photographs with the group and discuss their meaning.

### Demographic Characteristics

For phase 1, a total of 197 people participated with consent; however, demographic data were completed by only 130 (66%) participants (Table 1). The participants had a mean age of 65.4 years (SD 12.1 years, range 31-90 years), with a large proportion of older adults. Almost all participants were born outside of Canada, with most of them residing in the country for at least 10 years. Most of the participants (111/130, 85.4%) indicated that their physician was their primary source of health information, and approximately half reported that the internet, family and friends, and television and radio were secondary sources.

**Table 1:** Phase 1 participants’ demographics by language group (N=130)^a^.

Demographics		Punjabi (n=45)	Hindi (n=47)	Urdu (n=23)	Tamil (n=13)	Total (N=130)
**Age (years), n (%)**						
	<50	6 (13)	0 (0)	2 (9)	9 (75)	19 (15)
	51-60	2 (4)	4 (9)	2 (9)	0 (0)	8 (6)
	61-70	14 (31)	25 (53)	10 (46)	3 (25)	52 (41)
	>70	23 (51)	18 (38)	8 (36)	0 (0)	49 (38)
	Missing	0	0	1	1	2
**Gender, n (%)**						
	Female	27 (60)	23 (55)	7 (32)	6 (46)	64 (52)
	Missing	0	5	1	0	6
**Education, n (%)**						
	Secondary or below	15 (34)	11 (25)	10 (50)	1 (8)	37 (30)
	Diploma	4 (9)	10 (23)	4 (20)	1 (8)	19 (16)
	Undergraduate	17 (39)	12 (27)	3 (15)	4 (33)	36 (30)
	Postgraduate	8 (18)	11 (25)	3 (15)	6 (50)	30 (25)
	Prefer not to say	1	3	3	1	8
**Household income per year (CAD $; US $), n (%)**						
	<40,000 (31,241.10)	12 (27)	21 (57)	13 (87)	3 (38)	49 (62)
	40,000-60,000 (31,241.10-46,861.60)	4 (22)	9 (24)	1 (7)	0 (0)	15 (19)
	>60,000 (46,861.60)	2 (11)	7 (19)	1 (7)	5 (63)	15 (19)
	Missing or prefer not to say	27	10	8	5	48
**Place of birth, n (%)**						
	Born outside of Canada	43 (98)	46 (100)	23 (100)	13 (100)	127 (99)
	Born in Canada	1 (2)	0 (0)	0 (0)	0 (0)	2 (2)
	Missing	1	1	0	0	2
**Resident in Canada (years), n (%)**						
	<5	3 (7)	0 (0)	2 (9)	4 (31)	9 (7)
	5-10	5 (11)	5 (11)	3 (13)	3 (23)	16 (13)
	11-20	8 (18)	9 (20)	6 (26)	3 (23)	28 (22)
	>20	28 (64)	31 (69)	12 (52)	3 (23)	74 (58)
Participated in phase 2, n (%)		5 (11)	4 (9)	1 (4)	2 (15)	12 (9)

^a^Percentages were calculated after removing missing and prefer not to say responses from the denominator. In all, 2 participants included in the Total column were missing their language group.

### Themes

The qualitative analysis of the focus groups revealed various themes that were consistently identified in both phases. Reported here are first the themes common to both phases, followed by the themes unique to each phase. Finally, additional themes that were specific to phase 2 regarding the photovoice component are reported. Table 2 presents a summary of the core themes identified.

**Table 2:** Summary of barriers and facilitators to accessing digital health tools.

Summary	Facilitators	Barriers
Common themes across both phase 1 and phase 2	Social support (*eg, from family members to use digital health tools*)^a^ Positive attitude toward using digital health *Subthemes for positive attitudes*: Convenience (*eg, saves travelling time*) Enhances awareness of health (*eg, monitoring activity levels through wearables*) Reduces need for hospitalization (*eg, sleep apnea machines can be used at home*) Provides opportunities To learn about health (*eg, reading on the internet about how to manage health conditions*) For social connection (*eg, using Skype to connect with friends and family*) For physical activity and mental stimulation (*eg, technology use for games and activities*)	Language (*eg, English as a second language*) Literacy levels (*eg, medical terminology*) Computer and technology literacy (*not having learned to use computers*) Recognition of a negative impact on health (*associating time spent on the internet as being unhealthy*) Time constraints (*lack of time to learn to use digital health tools*)
Unique to phase 1 (focus groups only)	Familiar platforms and places (*for learning and knowledge sharing*)	Age-related self-efficacy (*lack of confidence in perceived ability to learn because of age*) Gender roles (*eg, women reporting less digital health tool use because of caring and familial responsibilities*) Limited trust in digital health sources (*many only trusting physicians’ word*) Cultural norms of communication (*preference for in-person interaction*)
Unique to phase 2 (photovoice)	Computer classes (*willingness to learn starting with computer classes*) Usability of technology (*accessibility and user-friendly technology encouraged digital health tool use*) Social sharing (*of health information has potential to promote use of digital health tools*)	Financial (*many reported financial constraints to accessing tools*) General self-efficacy (*general lack of confidence reported in ability to use tools*) Lack of motivation (*some reported a lack of desire to put effort into trying to use tools*) Lack of awareness (*many reported not knowing much about the tools available and how to access them*)

^a^Italicized results are further described in the subsections below.

### Common Facilitators Across Both Phases

#### Social Support

Support from family, especially younger generations, was frequently cited as a facilitator to using digital health tools: “ Online...I [get] help from my kids. I can’t use [a] laptop, but [my] kids always help me whenever I need to know something.” There was agreement across many participants that family is a great source of support in using technology: “[I] think most grandchildren, sons and daughters all provide us help in that case.”

#### Positive Attitude Toward Using Digital Health

In phase 1, participants identified more than 20 advantages for using digital health tools, for example:

It is very useful for health. It is good for general knowledge. You can see the whole world through [the] internet. It is a need of today’s life.

What I have noticed is after coming here from India, people are becoming more active physically and mentally. Only technology has made them more active.

Despite the challenges identified, participants largely agreed on the importance of technology, especially for those who have limited mobility: “To learn [the] internet is very necessary for those seniors who have difficulty in moving around.”

**Figure 1:**
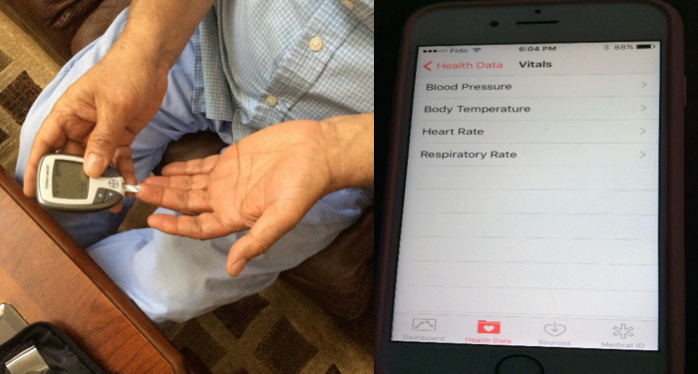
Photograph taken by a participant, with an example of a quote from a participant discussing this photograph in the focus group: “Now I can check my blood pressure at home whenever I have a need to do it...making it easy for us to check blood sugar, blood pressure, heart rate, etc.”

In phase 2, participants also identified numerous advantages to using digital health tools:

- Convenience: several participants photographed digital health tools such as pedometer devices, blood pressure machines, and blood glucose monitors (Figure 1) and talked about the convenience of health technology: “Now I can check my blood pressure at home whenever I have a need to do it....”
- Enhances awareness of health: discussion around the photograph of a Fitbit device sparked conversation about the power of technology to enhance awareness of health:

Awareness also [about] how much [exercise] you have done, how much you should do: it helps. If the steps are less [than they should be] you can walk more and if it’s more then you don’t exert anymore.

- Provides opportunities to learn about health: many participants referenced ways in which technology can provide opportunities to learn about health, for example:

If there is any health issues or disease, we can go to the internet and see why it has happened, the precaution we need to take and diet we need to follow. We can know everything from the internet.

Some participants spoke about the advantage of being able to read health information on the web in different languages:

If you bring up an article it will ask you...if you want to translate. When you say yes, it will give you options to choose the language.

- Reduces need for hospitalization: discussion around a photograph of a sleep apnea device revealed that several participants felt that technology plays an important role in reducing the need for hospitalization: “In the hospital somebody [with] more serious [illnesses] can get the bed and this patient got the facility at home.”
- Provides opportunities for social connection: another theme that arose was that technology can provide opportunities to connect with others:

Skype is a wonderful thing for lonely older people...when you want to talk to your relatives you can do that...you can see the person and have great satisfaction.

- Creates opportunities for physical activity and mental stimulation: another advantage referenced by several participants was the idea that technology can provide opportunities to be active both physically and mentally. For a participant, keeping mentally active played an important role in managing chronic pain: “It keeps me busy and keeps my mind off the pains I have.”

### Common Barriers Across Both Phases

#### Language

English was a second language for most participants. Even when language-specific resources were provided, participants expressed that the content was often either too academic or not properly translated, for example:

It’s not simple Punjabi. Complicated words are used in the translations.

**Figure 2:**
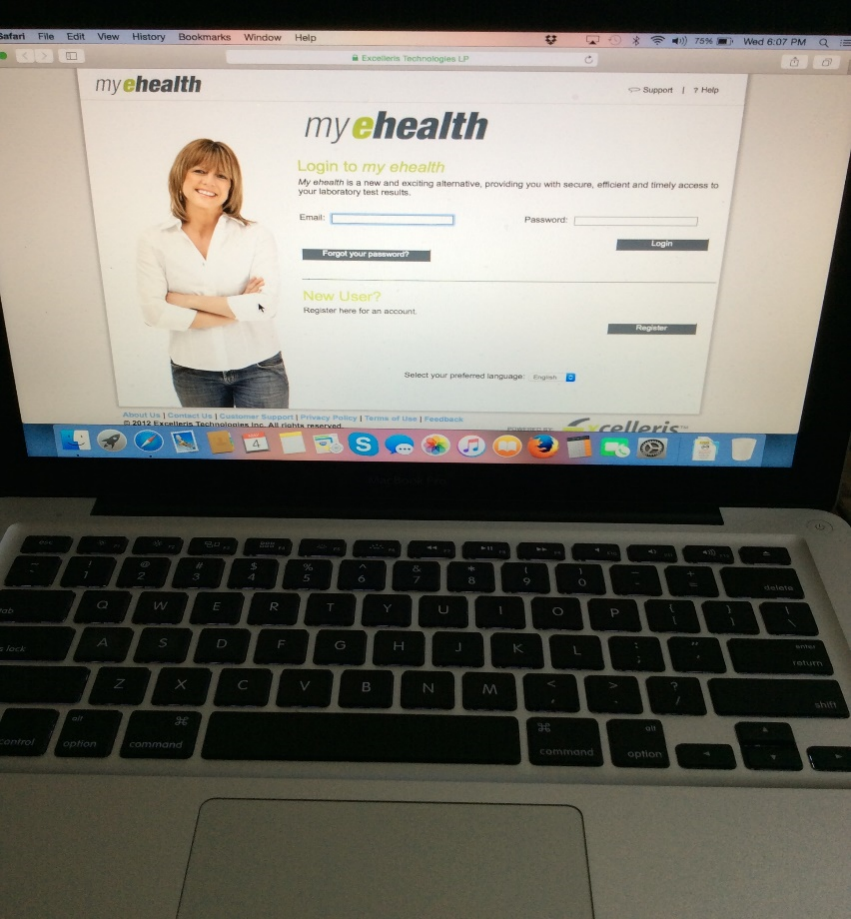
Photograph taken by a participant, with an example of a quote from a participant discussing this photograph in the focus group: “Though this picture does not show language, you go on internet and everything is in English, whereas it [is] not in their mother tongue. It is a challenge for many.”

Discussion around a photograph of a computer (Figure 2) also revealed these challenges:

You go on [the] internet and everything is in English, whereas it [is] not in their mother tongue. It is a challenge for many.

#### Literacy Levels

Many participants reported that they had difficulty reading in any language and therefore had a preference for radio or television:

If you tell me go to [the] internet and access the information but I am not educated, then how will I read that information? You send me literature; I can’t read it so it will go in the garbage. So, the best source is radio and TV.

#### Computer and Technology Literacy

Challenges in using technology and navigating web-based information were frequently cited: “I’m an illiterate when it comes to computers.” Some participants expressed an interest in learning to use computers, for example:

We don’t know how to use it but nobody even bothered, nobody tried to teach us, neither [did] we put [in the] effort to learn, but if somebody will teach us, we can learn.

#### Recognition of Negative Impact on Health

Some participants expressed awareness of the harmful side to excessive technology use: “There are many people who spend too much time on [the] internet and they don’t go outside for [a] walk....” Several participants in the photovoice phase noted that excessive technology use can lead to unhealthy behaviors, for example: “If we sit at the computer, three or four hours will go by easily, then later you realize that we have been focusing on only one thing and you have ignored many other things.”

#### Time Constraints

Time was also raised as a barrier to learning to use technology, especially for health: “Actually life is so busy that generally people are not available for sessions or for different aspects of technology especially in the health area...”

### Facilitators Unique to Phase 1

Familiar platforms and places*—*some participants discussed the importance of using familiar gathering places to increase the uptake of digital health tools:

To learn about health, in terms of motivating us [South Asian seniors], it has to be easy for them...If they go to community centers, gurdwaras, and other places, that would be the best places to teach the community.

As part of this conversation, some participants indicated that radio and television are ideal sources for information sharing in the community: “More information should be given through radio and TV.”

### Barriers Unique to Phase 1

#### Age-Related Self-efficacy

Concerns of being unable to learn new concepts or having poor memory were reported, which participants often attributed to age:

It’s of no use now...what [can I] learn at this age?

Some participants indicated that there is fear of learning for older community members:

For all the seniors, it is a phobia to use [the] internet or computer[s]. They think that they are 60+ and they cannot

learn anything new

#### Gender Roles

It was reported that because of women’s familial duties, they experienced more limited access to, and time for, seeking health information using digital technologies. Women reported using digital health tools less than men, often attributed to their workload and gender roles in the family: “[Women] have lots of household work as well as work outside [the house], too.” However, some participants also indicated that these gender differences in technology use are diminishing:

Most of the men use cell phones so they might be able to access [the] internet more than women. But it’s getting equal nowadays.

#### Limited Trust in Digital Health Sources

Physicians’ advice was considered by many participants to be more reliable than the internet or television and radio programs: “Because [my] doctor knows my diagnosis and can tell me the right things.” Some participants suggested that they felt it was important to corroborate health information they find on the web with their physician: “So whenever you get any kind of information, you must consult the doctor, before you go for it.” Many indicated that they felt that their physician was their primary source of health information: “[My] doctor is the ultimate source.”

#### Cultural Norms of Communication

Face-to-face interaction was preferred over internet-based communication among participants:

When email was newly introduced, my next door neighbor sent me an email about something. I felt like he slapped my face. I went to him and asked, “Can’t you talk to me about it? Why did you send me [that] in writing?”

### Facilitators Unique to Phase 2

#### Computer Classes

Many participants agreed that computer classes in the community would enable the use of health technologies: “We need some computer classes because we don’t want to depend on others.” Participants expressed that an important part of taking the classes is to spark people’s curiosity and focus on the interests of the community:

You have to start from somewhere. First of all they should start with what they are interested in.

One-to-one teaching was deemed more effective than group classes by participants, particularly for older adults in the community.

#### Usability of Technology

Several participants discussed the importance of technology being accessible and easy to use. A participant explained how this made a difference to his uptake of technology:

I didn’t know what the computer [was]. At that time, you didn’t have the easier ways. You didn’t have pointer. You couldn’t use [a mouse], you had to use certain keys to get into things...So I found it very difficult to learn at that time. Later on they found the easier ways.”

**Figure 3:**
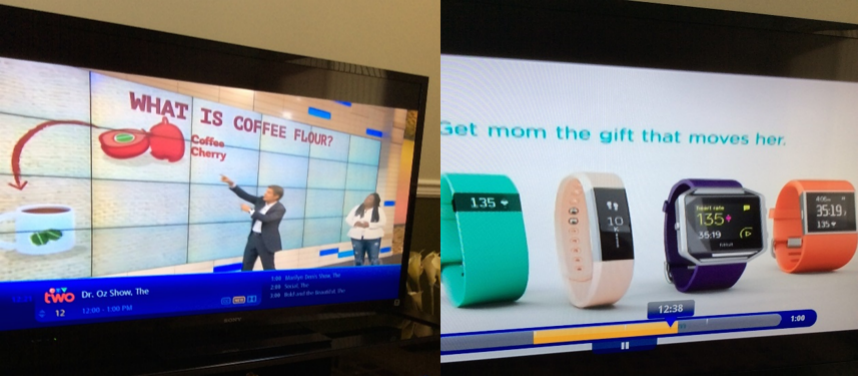
Photograph taken by a participant, with an example of a quote from a participant discussing this photograph in the focus group: “There are so many health channels on TV...there is exercise, diet, using equipment, and explain about technology...”

Furthermore, participants discussed ways in which media can be an accessible digital tool for improving health literacy in the South Asian community (Figure 3). Participants stressed that radio could be a particularly useful tool, especially for older adults:

Radio is almost in every senior’s home. If not at home then it is accessible on the phones these days.

#### Social Sharing

Another key facilitator to using technology for health was the cultural norm of social sharing among the South Asian community: “When somebody [hears] something [they] would tell another ten people.”

### Barriers Unique to Phase 2

#### Financial

Affordability was stated as a clear limiting factor for many people in the community to using technology for health: “If we want to get training ourselves, we don’t have money to spend, plus we also need time.”

#### General Self-efficacy

Another barrier discussed was the lack of confidence to use technology:

I think it’s a fear of confusion. Fear has to be overcome...removed. If you sit at the computer and don’t know what to do or how to start, then how can you use it? Then it is very intimidating.

#### Lack of Motivation

Motivation was also discussed as an obstacle, largely because of the effort required to learn:

They couldn’t be bothered. I know the advantages...they think there are some hassles, there is too much trouble to learn.

#### Lack of Awareness

Lack of awareness concerning which digital health tools are available. A participant stated as follows:

I think the greatest barrier is awareness. If people are not aware of what technology is there to manage their health, they will not use it.

### Experiences of Photovoice

#### Meaningful and Enjoyable Experience

Participants spoke about the photovoice phase itself as being a meaningful and enjoyable learning experience:

It was a good time. We had a chance to see many new things.

A total of 3 key themes emerged from the focus groups to capture the participants’ experiences.

#### Learning Opportunity

For many participants, using photovoice promoted learning about technology and health in general:

There was a lot of exploration...what options are available...how technology is helping in health.

More specifically, participants’ awareness of how technology can be used to promote health seemed to grow through the photovoice process:

I really liked it...With new technology, we come to know about so many things. We can come to know how to use technology and how it can help us.

#### Personal Growth

Many participants highlighted the novelty of the experience that led to self-reflection and personal growth:

The significant moment was when I was taking pictures and started thinking why I was taking the photographs. It opened a field for me. What was happening and why was it happening with me then. It was very enlightening for me.

Another participant stated as follows: “Actually we gained confidence with it.”

#### Encourages Healthy Behaviors

Among other benefits referenced, some felt it engaged them in a healthy mental activity:

We kept busy. We had [a] kind of brain exercise.

In addition, it encouraged physical activity among some participants: “We get a chance to go out and walk around.”

## Discussion

### Thematic Findings

This multiphase CBPAR study with the South Asian community in Surrey, British Columbia, Canada, investigated facilitators of and barriers to the uptake of digital health tools to prevent and manage chronic health conditions. Some of the barriers identified reflect recent research that immigrants, older adults, and older ethnic minority individuals are less likely to use digital health tools for their health care activities [20]. In addition, participants stated that age, gender, income, and education presented obstacles to their use of digital health tools, which closely echoes the findings from a study of Punjabi-speaking individuals in Metro Vancouver [12]. Perceived gender roles were cited as reasons why *women* may use digital health tools less than *men* because of familial and household duties; however, digital technology can save time and therefore can be a solution for those who have less time or more caring responsibilities. Low English and computer and technology literacies were salient themes and reinforce the need for digital health innovations to be linguistically appropriate and accessible to ethnic minority populations [12,19]. Interestingly, many individuals reported high educational levels (66/122, 54.1%) had an undergraduate or postgraduate degree); even so, a theme emerged around difficulty reading in any language (even first language). A possible explanation for this is that participants were referring more specifically to difficulty understanding medical language, which can be challenging for many people from different cultural and educational backgrounds, particularly considering that those who reported education up to undergraduate and postgraduate level were educated in their country of origin where English language proficiency is limited and often restricted to their professional areas. In addition, we are missing data for background education for 34% (67/197) of the participants; therefore, it could be that this theme represented those with lower levels of education.

A first step in promoting the use of digital health tools in the South Asian community may be to address levels of health and computer literacies. By improving digital health literacy, participants indicated that they would be more likely to start using digital health tools to manage their own health. This important point is intertwined with other themes elucidated, namely that promoting digital health literacy will also help participants to address barriers around their self-efficacy, motivation, and awareness. Community-based promotion efforts could incorporate elements of social sharing, social support, and cultural norms to improve the likelihood of success. Furthermore, there seemed to be some disconnect between the research team’s and the participants’ perspectives on the technology that constituted *digital health tools*. The participants took a broader view and discussed technology that affected their health, such as the use of videoconferencing for social connection. Thus, it may be necessary to establish a shared definition of *digital health tools* for research and promotion with South Asian communities as well as to focus research on specific digital health tools to be able to take more concrete steps toward improving accessibility of specific tools.

These findings highlight other barriers to the use of digital health tools, namely cultural norms, lack of trust, and time and financial constraints (ie, social determinants of health [SDoH]) [31]. These are important factors to consider and may explain some of the gaps in digital health access for South Asians in Canada. Interestingly, given the number of barriers identified, the participants also seemed to have a generally positive attitude toward digital health tools and were interested in learning more about them and their potential benefits. This reinforces the need for accessible, culturally sensitive digital health tools that are not prohibitively costly in terms of money or time investment. The ways in which tools may be considered culturally sensitive must be guided by the users themselves and not researchers or technology developers. However, this study indicates some ways in which this might be achieved, such as options for first language and perhaps adopting elements of social sharing that do not rely on both or all users accessing technology directly by incorporating a *buddy system*. This may mean that friends and family can benefit from the technology without necessarily having to navigate it directly but through the support of those who feel more confident. Although not specifically discussed in this study, cultural sensitivity must also consider language that may not be appropriate, for instance, language that is more representative of Westernized culture.

### The Value of a CBPAR Approach and Photovoice

Our findings support the validity and significance of using CBPAR methods, including photovoice, to develop and explore research questions with ethnocultural populations, while also building knowledge and skills in these communities. Although previous literature has highlighted the risk of CBPAR methods leading to tension between community members and researchers or the possible loss of research objectivity, we concur with experts that CBPAR is a strong approach for promoting health equity, especially in marginalized communities [32,33]. The photovoice participants reported that they increased their awareness of, and confidence in, using digital health tools, which was a success of the capacity-building component of the research initiative. Participants were able to learn about different aspects of health and digital tools through exploration; they had the opportunity to connect and learn with others, while being supported by research team members. The project built research capacity in the community by engaging and mentoring community leaders and PCRs to facilitate the research process, ensuring that all elements were language-appropriate, and making the study meaningful for the community. Although both the focus group and photovoice methods identify common themes, each method further contributed unique insights that provide a more holistic picture of the barriers to and facilitators of the uptake of digital health tools. Although we set out to explore the barriers to and facilitators of digital health tool use, this paper also highlights the challenges of conducting CBPAR in a way that minimizes the potential for bias.

### Limitations

Although a large sample was recruited from Surrey’s South Asian community, there may be limited generalizability to the wider community of South Asian individuals residing in Canada. For example, South Asian Canadians living in more rural settings or serviced by different regional health care systems may experience other barriers to accessing digital health literacy. Most participants spoke Punjabi or Hindi, the 2 dominant South Asian languages spoken in Surrey [27]. Further work could elaborate on any issues specific to the Urdu- and Tamil-speaking communities (or other minority language groups) as well as to understand how facilitators and barriers may differ among cultural groups or by other demographics. In addition, demographic data beyond language group were missing for a subset of participants, thereby limiting our ability to speculate on the link between the themes and characteristics of the whole sample. The photovoice phase met the gold standard for sample size by involving 12 individuals [23,24]; however, multiple rounds of photovoice with more participants may have gleaned additional information and themes. In addition, we are not able to report on the demographic characteristics of the photovoice participants, thus potentially overlooking deeper gender-based insights. Furthermore, a deeper form of analysis such as discourse analysis could have been valuable to explore any influences that could come from the agenda of community leaders potentially biasing participant responses.

### Implications for Policy and Future Work

Considering the increasing prevalence of digital health tools within the current health care landscape, these findings highlight considerations for the use of these tools among South Asian Canadians in terms of accessing health information and managing health conditions and preventing chronic disease. On the basis of this study’s findings and past work, there seem to be several policy and health care recommendations, which are organized here according to level [31]:

- Microlevel (patients and caregivers)
  Continue to assess the current and future health needs of ethnic minority groups accessing health care, including digital health literacy and SDoH.
  Involve patients and caregivers throughout research and health initiatives to ensure that these efforts are meaningful and culturally appropriate.
- Mesolevel (community)
  Evaluate community attitudes toward digital health tools to inform optimal approaches in implementing new digital health initiatives.
  Integrate CBPAR approaches and photovoice as methods of inquiry to ensure that diverse and holistic perspectives are collected [24,26].
  Incorporate cultural norms and preferences into initiatives to develop and promote digital health literacy to maximize their appeal and accessibility.
  Develop versions of digital health tools that are not prohibitively costly in terms of money or time investment.
- Macrolevel (education and health system)
  Engage community leaders, health care providers and administrators, and technology developers to better understand the needs of groups with varying SDoH.
  Raise awareness among health care providers regarding facilitators and barriers around digital health tool uptake, with the objective of improving providers’ communication and prescription of digital tools to patients.
  Provide opportunities for health professionals and trainees to learn from multicultural patient and community populations to develop greater understanding of potential barriers and cultural considerations.

### Conclusions

In the transformation of health systems to introduce digital technology innovations, it is necessary to support multicultural populations and to prevent paradoxical development of health inequity for those who have difficulties using digital tools to access health information or services [34]. This CBPAR study revealed key barriers and facilitators related to digital health tool uptake among this sample of South Asian Canadians. The findings emphasize the need to overcome language and cultural barriers for meaningful engagement and prioritize community participation to get to the key issues. Future health promotion strategies and research should consider these methods and findings to ensure that community members with different cultural and language backgrounds have the opportunity to inform the development and use of effective, culturally appropriate digital health tools.

## Abbreviations

CBPAR: community-based participatory action research
iHEAL: Interactive Health Education Action for Life
PCR: peer community researcher
SDoH: social determinants of health
